# Transactions of the Academy of Sciences at Sienna

**Published:** 1782

**Authors:** 


V. *Atti dell' Academia delle Scienze di Siena*: i. e *Transactions of the Academy of Sciences at Sienna*. Vol. v. 4to. Sienna, 400 pages, with four copper plates.

O F

OF the papers contained in this volume, only the two following come within the plan of our journal:

1. *An account of a pure, native, concrete vitriolic acid. By* Jofeph Baldaffari. The fubftance here defcribed was found by our author in a cave near the hot baths of St. Philip, in the neighbourhood of Sienna. The fprings that fupply thefe baths iffue from a mountain, which from its abounding with lava and other volcanic productions, is with good reafon fuppofed to have been formerly a volcano. The bottom of the cave is covered with a native chryftallized fulphur, and the fides and top of the cave, afford minute, white chryftals which tafte like fpirit of vitriol, and prove to be a pure vitriolic acid.

2. *An account of a reputed hermaphrodite. By* Francis Caluri.—Auguftus Broli, the perfon who is the fubject of this paper, and who was faid to be an hermaphrodite, having been examined by our author, was found to have a fmall penis with an imperforated prepuce. There was no appearance of tefticles externally, and inftead of a fcrotum, the cutis formed a ridge furnifhed with a foramen through which urine and, occafionally, femen were difdischarged. In other respects he had all the appearance of a male, except that instead of a beard he had only a soft down. From the author's account it seems clearly to appear that this person, like all those who have hitherto been described as hermaphrodites, is in reality a male with some malconformation of the organs of generation.

